# Heat and mass transfer analysis of assisting and opposing radiative flow conveying ternary hybrid nanofluid over an exponentially stretching surface

**DOI:** 10.1038/s41598-023-41916-6

**Published:** 2023-09-08

**Authors:** K. V. Nagaraja, Umair Khan, J. K. Madhukesh, Ahmed M. Hassan, B. C. Prasannakumara, Nabil Ben Kahla, Samia Elattar, Jasgurpreet Singh Chohan

**Affiliations:** 1 Department of Mathematics, Amrita School of Engineering, Amrita Vishwa Vidyapeetham, Bengaluru, 560035 India; 2 Department of Mathematical Sciences, Faculty of Science and Technology, Universiti Kebangsaan Malaysia, UKM Bangi, 43600 Selangor, Malaysia; 3 Department of Computer Science and Mathematics, Lebanese American University, Byblos, Lebanon; 4 Department of Mathematics and Social Sciences, Sukkur IBA University, Sukkur, 65200 Sindh Pakistan; 5 Mechanical Engineering, Future University in Egypt, New Cairo, 11835 Egypt; 6 Department of Studies in Mathematics, Davangere University, Davangere, 577002 India; 7 Department of Civil Engineering, College of Engineering, King Khalid University, Abha, 61421 Kingdom of Saudi Arabia; 8 Department of Industrial & Systems Engineering, College of Engineering, Princess Nourah bint Abdulrahman University, P.O. Box 84428, Riyadh, 11671 Saudi Arabia; 9 Department of Mechanical Engineering and University Centre for Research & Development, Chandigarh University, Mohali, Punjab 140413 India

**Keywords:** Mathematics and computing, Applied mathematics, Chemical engineering

## Abstract

Access to dependable and environmentally friendly energy sources is critical to a country's economic growth and long-term development. As countries seek greener energy alternatives, the interaction of environmental elements, temperature, and sunlight becomes more critical in utilizing renewable energy sources such as wind and bioenergy. Solar power has received much attention due to extraordinary efficiency advances. under this context, the present work focus on solar radiation and chemical processes in the presence of modified ternary hybrid nanofluids (THNFs) circulating over an exponentially stretched surface in both aiding flow (A-F) and opposing flow (O-F) circumstances. The primary objective of this investigation is to dive into the complicated dynamics of these structures, which are distinguished by complex interactions involving radiation, chemical reactions, and the movement of fluids. We construct reduced ordinary differential equations from the governing equations using suitable similarity transformations, which allows for a more in-depth examination of the liquid's behavior. Numerical simulations using the Runge–Kutta Fehlberg (RKF) approach and shooting techniques are used to understand the underlying difficulties of these reduced equations. The results show that thermal radiation improves heat transmission substantially under O-F circumstances in contrast to A-F conditions. Furthermore, the reaction rate parameter has an exciting connection with concentration levels, with greater rates corresponding to lower concentrations. Furthermore, compared to the O-F scenario, the A-F scenario promotes higher heat transfer in the context of a modified nanofluid. Rising reaction rate and solid fraction volume enhanced mass transfer rate. The rate of thermal distribution in THNFs improves from 0.13 to 20.4% in A-F and 0.16 to 15.06% in O-F case when compared to HNFs. This study has real-world implications in several fields, including developing more efficient solar water heaters, solar thermal generating plants, and energy-saving air conditioners.

## Introduction

Science and advanced methods as well as recent technology have played a prominent role in the production of power^1^, refrigeration and heating^2^, production^3^, applications in medicine^4^, pharmaceutical industries^5^. To operate effectively and functionally of these systems will depends on excellent thermal management systems^6^. Further, improving the power efficiency and minimizing the components will challenge the existing techniques. To overcome this a new concept of enhancing the thermal conductivity in working fluids by involving micro-scale nanoparticles was introduced by Hamilton and Crosser^7^. In 1995, Choi and Eastman^8^ conducted pioneering research on using nanoparticles (NPs) to improve liquid thermal conductivity. The resulting liquid formed is coined as nanofluid (NFs). These fluid shows gradual improvement in its thermal conductivity due to this NFs applications are found in^9–12^. Based on the applications, we can find some of the advantages of nanofluids with relevant to base liquid. The rate at which heat is exchanged in thermal systems may be increased by utilising a nanofluid because of its better thermal conductivity relative to the base liquid. Therefore, by employing nanofluids to increase the temperature transfer rate, the size of a thermal system may be lowered, meaning a more compact system with savings in material weight and cost. Nanofluids are more stable and can improve the transmission of heat more than carrier fluids can. In view of this, numerous researchers have examined and evaluated the usage of nanofluids in thermal transfer applications.

Sandeep et al.^13^ analyzed the flow characteristics of a chemically reactive Casson liquid over a convectively heated curved area. The results demonstrate that non-Newtonian fluids, in contrast to Newtonian fluids, have a much higher heat transfer rate under unequal heat sources and viscous dissipation conditions. Yasir et al.^14^ looked at the dynamics of ethylene glycol transporting copper and titania NPs over a stretchable/shrinkable curved structure, including a stability in their study. Because copper enhances porosity and titania functions as a photocatalyst, the study also emphasises the significance of these nanocomposites. Prasannakumara et al.^15^ explored the TPD (Thermophoretic particle deposition) in a bioconvective NFs flowing over an exponentially extended surface. The findings reveal that adding NPs in a carrier fluid will increases the heat transformation rate. Poojari et al.^16^ evaluated improved conveyance of heat in unsteady magneto- NFs circulation caused by a stochastic expanding surface with convective boundaries. They employed the Maxwell and Xue models of NFs in their research. According to the results, the Maxwell nano model predicts a far higher rate of thermal circulation than the Xue nano model predicts. Khan et al.^17^ evaluated the effect of mixed convection and radiation on the temperature transfer of a nanofluid in a slip flow through a bending sheet subject to activation energy and a binary reaction. Results reveal that the addition of nanoparticles slows the flow rate for both upper and lower branch solutions. Some of the works with different kinds of nanoparticles in base liquds are listed in^18–21^.

A specialised group of NFs known as HNFs (hybrid nanofluids) has arisen in recent years. An innovative kind of nanofluid, HNFs are made by dispersing many NP types in a single solvent. A specific material may not contain all desirable features necessary for a certain application; it may have excellent thermal or rheological capabilities. An owing to the synergistic effect, the HNFs is projected to have higher thermal conductivity than separate nanofluids. Recently, Nanda et al.^22^ studied the nonlinear/linear expandable surface containing joule impact, radiation with three dimensional movement of tangent hyperbolic liquid containing aluminium alloys. The influence of nanoparticles, the heat transfer rate in the nonlinear extended scenario is double that of the linear stretched case. Ramesh et al.^23^ examined the HNFs circulation across a nonlinear/linear stretched surface by considering TPD. In the study, linear situation, including nanoparticles augments heat propagation but decreases concentration while improving axial velocity in the nonlinear case. Yasir et al.^24,25^ explored the thermal performance of different kind of nanoparticles (SWCNT, CuO, MgO, Ag) with H_2_O and EG carrier liquids. Sulochana et al.^26^ examined the tangent hyperbolic liquid containing Mgo and Cuo nano sized particles in the presence of magnetic field. In the nanoparticle volume factor, the energy transmission rate in the nonlinear extending case is greater than the linear case.

Researchers are also investigating the inclusion of different NPs into HNFs, resulting in modified nanofluids. This novel class of functional liquids shows promise and is the topic of much investigation. Trihybrid nanofluids (TNFs), made up of three solid NPs combined with a carrier liquid, have been studied for their improved thermal conductivity and thermal expansion. Recently, Yook et al.^27^ used a multi-linear regression model to examine ternary hybrid nanoparticles' thermal and momentum transmission in a channel with varying permeabilities and porous walls. In their study they used three different cases of nanoparticle combination to examine the thermal performance. The results show that more rapid heat transmission is shown in mixtures of paraffin wax, sand, and AA7072. Madhukesh et al.^28,29^ examined the TNFs flow to examine the thermal and mass transfer analysis. The TNFs shows greater performance in these two aspects than HNFs and NFs. Animasaun et al.^30^ studied thermal examination of magnitudes of different kinds of nanoparticles with water as a base liquid. More thermal significant performance is achieved with smaller densities of the nanoparticles.

Aerodynamics, plastic sheets preparation, processing of materials, wire drawing and environmental flows are some of the examples which draw the attention of exponential stretching sheet (E-SS). The study also draws an attention the impacts of this geometry and the movement of the surface on motion of the fluid and thermal distribution. Due to this many works are carried out on E-SS geometries. Prasannakumara et al.^15^ investigated the TPD, H-SS and movement of microorganisms over an E-SS using NFs. Alqahtani et al.^31^ examined a change in energy and mass due to the motion of a Casson hybrid nanofluid over an elongated stretching sheet. In their study thermal and slippage of velocity circumstances, the absorption of heat, viscous dissipation, thermal radiation, the Darcy impact, and thermophoresis diffusion impacts are studied. Carreau flow of Cu-water nanofluids through an exponentially permeable stretched thin surface with an MHD thermal boundary layer is quantitatively studied by Yousif et al.^32^. Souayeh and Ramesh^33^ studied the mobility of metallic ternary nanofluids (Ag–Au–Cu/H_2_O), taking into account a wide variety of phenomena like gyrotactic organisms, energy of activation, buoyancy forces, and thermal radiation, as they enacted through an exponentially extending sheet.

The mechanics of fluids and the transfer of heat combine at the interface, where thermal radiation plays a crucial role in determining liquid behavior. Without a medium, heat may be transferred from one place to another by means of electromagnetic waves; this process is governed by thermal radiation (T-R). Intricate studies are required to effectively characterize and forecast liquid behavior when T-R is included in the governing equations. To create effective thermal systems, such as solar panels, burning chambers, or spacecraft re-entry scenarios, it is necessary to have a firm grasp of these interactions. Yasir et al.^34^ looked into the effects of non-linear T-R and non-uniform H-SS on the movement of ethylene glycol in the presence of hybrid nanomaterials like Silicon dioxide and Titanium oxide. The results indicate that in both variants of the optimal solution, heat transmission improves as a result of an increase in the radiation effect. The effect of electromagnetic radiation and convective slippery conditions on second-grade nanofluids with high viscosity via permeable medium was studied by Jamshed et al.^35^. The study reveals that increasing radiation levels modify its temperature distribution and the Nusselt number. The consequences of heat production and absorption in the T-R mixed convective circulation of a hybrid nanofluid through an inclinically contracting interface were studied by Yasir et al.^36^. the study reveals that boosting thermal dispersion through a rise in T-R and Eckert numbers. Some recent works on T-R with TNFs are given in^37,38^.

In a chemical reaction, two or more chemicals undergo a transformation to produce a new compound. Chemical reactions (C-R) form the backbone of the discipline and perform an essential role in many other areas of science, industry, and daily life. They play a role in everything from combustion to metabolism to photosynthesis to the creation of new materials. The areas of health, materials research, energy generation, and environmental protection all stand to benefit greatly from a deeper understanding of, and ability to manipulate, chemical interactions. Khan et al.^39^ examined the time-dependent movement of non-Newtonian fluid, emphasizing thermal and solutal movement. The results demonstrate that an upsurge in the surface concentration of the catalyst enhanced the efficiency of both homogeneous and heterogeneous techniques. The impact of endothermic/exothermic C-Rs with activation energy on a wedge-shaped ternary hybrid nanofluid was studied by Sajid et al.^40^. The study reveals that C-R will upsurges rate of thermal distribution. Using a squeezed parallel infinite plate as a flow channel, Bilal et al.^41^ studied the characteristics of a fluctuating electroviscous TNFs. The study shows that as C-R values rise, the pace at which mass is allocated rises.

In all the above served literatures, the studies individually examined the impacts of T-R, chemical reaction, and porous medium over different geometries. No work is found to be studied by integrating all the above-mentioned impacts over E-SS in the presence of TNFs. Analyzing the resultant complicated system of ordinary differential equations requires the use of innovative numerical techniques like the Runge–Kutta Fehlberg (RKF) method and shooting algorithms. The major engineering coefficients are also covered in this study. The results have real-world applications in areas such as thermal engineering, manufacturing processes, and renewable energy infrastructure. Finally, the aim of the article provides answers to the following pertinent research questions:

- How do several important variables affect both A-F and O-F cases?
- What effect do changes in various parameters have on the rate of heat transfer and skin friction?
- How change in chemical reaction constraint will interact with mass transfer rate?

## Mathematical modelling

**Figure 1:**
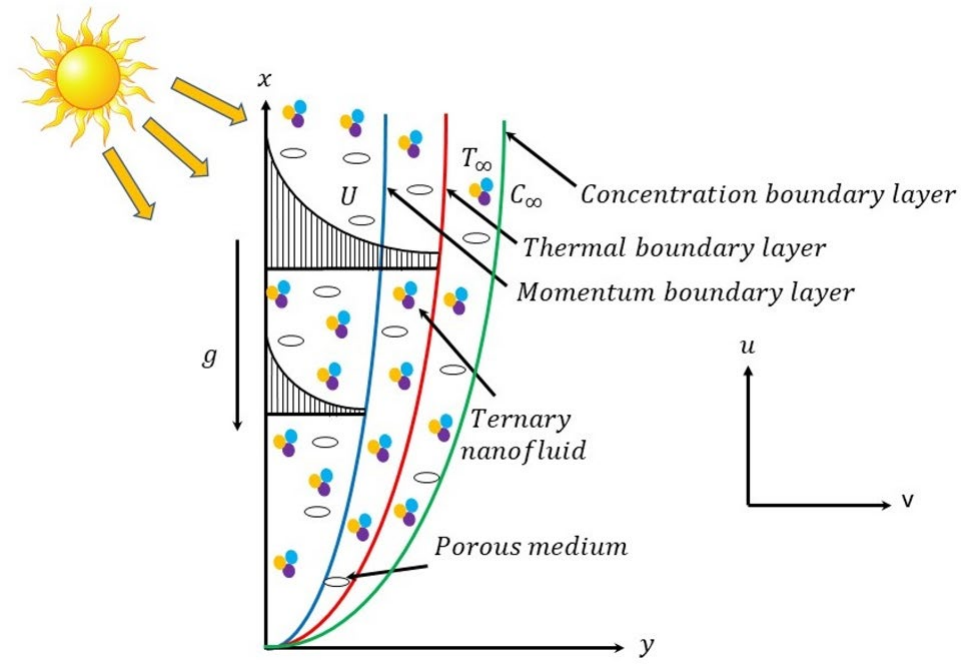
Geometric representation of the problem.

The steady, two-dimensional, laminar and incompressible flow of THNF (combination of ${\text{Al}}_{2} {\text{O}}_{3} + {\text{TiO}}_{2} + {\text{Ag}}$ nanoparticles and $H_{2} O$ base fluid), see Fig. 1. The sheet is stretching along *x*-direction and *y*-axis normal to it. The scenario of mutual assistance between the stretching-generated flow, thermally buoyant flow, and as well as the scenario of mutual antagonism between these two flows are studied. The sheet uniform velocity is represented by $U = U_{*} e^{\frac{x}{l}}$ in the corresponding $x -$ axis direction. Further, the sheets variable temperature is provided by $T_{w} = T_{\infty } + T_{0} e^{\frac{x}{2l}}$. Here, $T_{w}$,$T_{\infty }$ and $T_{0}$ denotes the wall, ambient and reference temperature of the sheet, respectively. It is also assumed that the variable concentration of the sheet is $C_{w} = C_{\infty } + C_{0} e^{\frac{x}{2l}}$. In which; $C_{w}$,$C_{\infty }$ and $C_{0}$ denotes the respective wall, ambient and reference concentration of the exponential surface of the sheet. However, $\delta_{r}$ is the variable chemical rate introduced into concentration equation^42^ and it is defined as $\delta_{r} = 0.5 \times \delta_{0} e^{\frac{x}{l}}$. Meanwhile, at $y = 0$, the temperature and concentration at the surface of E-SS is denoted by $T_{w}$ and $C_{w}$, respectively. The far field boundaries as $y \to \infty$ are presented by the respective $T_{\infty }$ and $C_{\infty }$. Based on these assumptions with boundary layer and Boussinesq approximations, the governing equations for the present problem are as follows: (see^43–46^):

1 $$\frac{\partial u}{{\partial x}} + \frac{\partial v}{{\partial y}} = 0,$$

2 $$u\frac{\partial u}{{\partial x}} + v\frac{\partial u}{{\partial y}} = \nu_{mnf} \frac{{\partial^{2} u}}{{\partial y^{2} }} - \frac{{\nu_{mnf} }}{{K^{*} }}u \pm \frac{{g\left( {\beta \rho } \right)_{mnf} }}{{\rho_{mnf} }}\left( {T - T_{\infty } } \right),$$

3 $$u\frac{\partial T}{{\partial x}} + v\frac{\partial T}{{\partial y}} = \frac{{k_{mnf} }}{{\left( {\rho C_{p} } \right)_{mnf} }}\frac{{\partial^{2} T}}{{\partial y^{2} }} - \frac{1}{{\left( {\rho C_{p} } \right)_{mnf} }}\frac{{\partial q_{r} }}{\partial y},$$

4 $$u\frac{\partial C}{{\partial x}} + v\frac{\partial C}{{\partial y}} = D_{B} \frac{{\partial^{2} C}}{{\partial y^{2} }} - \delta_{r} (C - C_{\infty } ).$$

The boundary conditions (BCs) are

5 $$u = U,v = 0,T = T_{w} ,C = C_{w} \;\;{\text{at}}\;\;y = 0,$$

6 $$u \to 0,T \to T_{\infty } ,C \to C_{\infty } \;\;{\text{as}}\;\;y \to \infty .$$

The last term in Eq. (2) specifies the effect of buoyancy force which has a negative and positive sign, where this positive sign refers the buoyancy assisting flow and the negative sign refers the case of buoyancy opposing flow. Moreover, in Eq. (3) the term $q_{r}$ is given by $q_{r} = - \frac{{4\sigma^{*} }}{{3k^{*} }}\frac{{\partial T^{4} }}{\partial y}$ (Rosseland approximation), see^47^. Additionally, expressing the term $T^{4}$ as a linear function of $T$ and ignoring the higher order terms, we get $T^{4} = 4T_{\infty }^{3} T - 3T_{\infty }^{4}$. Finally, the equation become as $q_{r} = - \frac{{16\sigma^{*} }}{{3k^{*} }}T_{\infty }^{3} \frac{\partial T}{{\partial y}}$. By using the below suitable similarity (see^44^):

7 $$\left. \begin{gathered} \eta = ye^{\frac{x}{2l}} \sqrt {\frac{{U_{*} }}{{2l\nu_{f} }}} ,\Psi = \sqrt {2\nu_{f} lU_{*} } e^{\frac{x}{2l}} f\left( \eta \right),\chi = \frac{{C - C_{\infty } }}{{C_{w} - C_{\infty } }} \hfill \\ \theta = \frac{{T - T_{\infty } }}{{T_{w} - T_{\infty } }},u = e^{\frac{x}{l}} U_{*} f^{\prime},v = - \sqrt {\frac{{\nu_{f} U_{*} }}{2l}} e^{\frac{x}{2l}} \left( {\eta f^{\prime} + f} \right) \hfill \\ \end{gathered} \right\}.$$

Using Eq. (7) in Eqs. (2–4), one obtains the form as:

8 $$f^{\prime \prime \prime } + A_{1} A_{2} \left( {ff^{\prime \prime } - 2f^{\prime 2} } \right) - K_{1} f^{\prime } \pm \lambda A_{1} A_{4} \theta = 0,$$

9 $$\frac{1}{{A_{3} \Pr }}\left( {\frac{{k_{mnf} }}{{k_{f} }} + \frac{4}{3}Rd} \right)\theta^{\prime \prime } + \theta^{\prime } f - f^{\prime } \theta = 0.$$

10 $$\chi^{\prime \prime } + Sc[f\chi^{\prime } - Rr\chi ] = 0,$$

with boundary conditions are:

11 $$f^{\prime } \left( 0 \right) = \chi \left( 0 \right) = \theta \left( 0 \right) = 1,f\left( 0 \right) = 0,$$

12 $$f^{\prime } \left( \infty \right) = \theta \left( \infty \right) = \chi \left( \infty \right) = 0.$$

From the above equations the non-dimensionless parameters are tabulated in Table 1.

**Table 1:** List of dimensionless influential parameters.

Sl. No	Symbol	Name of the parameter	Special case
01	$\lambda = \frac{Gr}{{{\text{Re}}^{2} }} = \frac{{g\beta T_{0} l}}{{U_{*}^{2} }}$	Mixed convection parameter	$\lambda < 0$ O-F case $\lambda > 0$ A-F case
02	$\Pr = \frac{{\nu_{f} \left( {\rho C_{p} } \right)_{f} }}{{k_{f} }}$	Prandtl number	
03	$Rd = 4T_{\infty }^{3} \frac{{\sigma^{*} }}{{k^{*} k_{f} }}$	Radiation parameter	
04	$Rr = \frac{{\delta_{r} l}}{{U_{*} }}$	Reaction rate parameter	
05	$Sc = \frac{{\nu_{f} }}{{D_{B} }}$	Schmidt number	
06	$K_{1} = \frac{{\nu_{f} l}}{{U_{*} K^{*} }}$	Porous permeability constraint	
07	$A_{1} = \left( {1 - \left( {\Lambda_{1} + \Lambda_{2} + \Lambda_{3} } \right)} \right)^{2.5}$		
08	$A_{2} = \left( {1 - \Lambda_{1} - \Lambda_{2} - \Lambda_{3} } \right) + \Lambda_{1} \left( {\frac{{\rho_{pS1} }}{{\rho_{f} }}} \right) + \Lambda_{2} \left( {\frac{{\rho_{pS2} }}{{\rho_{f} }}} \right) + \Lambda_{3} \left( {\frac{{\rho_{pS3} }}{{\rho_{f} }}} \right)$		
09	$A_{3} = \left( {1 - \Lambda_{1} - \Lambda_{2} - \Lambda_{3} } \right) + \Lambda_{1} \left( {\frac{{\rho_{S1} C_{pS1} }}{{\rho_{f} C_{pf} }}} \right) + \Lambda_{2} \left( {\frac{{\rho_{S2} C_{pS2} }}{{\rho_{f} C_{pf} }}} \right) + \Lambda_{3} \left( {\frac{{\rho_{S3} C_{pS3} }}{{\rho_{f} C_{pf} }}} \right)$		
10	$A_{4} = \left( {1 - \Lambda_{1} - \Lambda_{2} - \Lambda_{3} } \right) + \Lambda_{1} \left( {\frac{{\rho_{S1} \beta_{pS1} }}{{\rho_{f} \beta_{f} }}} \right) + \Lambda_{2} \left( {\frac{{\rho_{S2} \beta_{pS2} }}{{\rho_{f} \beta_{f} }}} \right) + \Lambda_{3} \left( {\frac{{\rho_{S3} \beta_{pS3} }}{{\rho_{f} \beta_{f} }}} \right)$		

Nanofluid and its correlations are discussed by many researchers. The first model of thermal conductivity of the nanofluids proposed by Maxwell model^48^. Later, Yu and Choi^49^ considered the nanoliquid layer and proposed new thermal conductivity. By considering Brownian motion and aggregation, Xuan^50^ proposed new thermal conductivity of the nanoliquids. Some of the other thermophysical correlations of the nanofluids are given in the works of^4,51–53^.

Thermophysical properties of TNFs used in the present study are given as follows (see^54^)

$$\mu_{mnf} = \frac{{\mu_{f} }}{{\left( {1 - \left( {\Lambda_{1} + \Lambda_{2} + \Lambda_{3} } \right)} \right)^{2.5} }}$$

$$\rho_{mnf} = \rho_{f} \left( {\left( {1 - \Lambda_{1} - \Lambda_{2} - \Lambda_{3} } \right) + \Lambda_{1} \left( {\frac{{\rho_{S1} }}{{\rho_{f} }}} \right) + \Lambda_{2} \left( {\frac{{\rho_{S2} }}{{\rho_{f} }}} \right) + \Lambda_{3} \left( {\frac{{\rho_{S3} }}{{\rho_{f} }}} \right)} \right)$$

$$\left( {\rho C_{p} } \right)_{mnf} = \left( {\rho C_{p} } \right)_{f} \left( {\left( {1 - \Lambda_{1} - \Lambda_{2} - \Lambda_{3} } \right) + \Lambda_{1} \left( {\frac{{\rho_{pS1} C_{pS1} }}{{\rho_{f} C_{pf} }}} \right) + \Lambda_{2} \left( {\frac{{\rho_{pS2} C_{pS2} }}{{\rho_{f} C_{pf} }}} \right) + \Lambda_{3} \left( {\frac{{\rho_{pS3} C_{pS3} }}{{\rho_{f} C_{pf} }}} \right)} \right)$$

$$\left( {\rho \beta } \right)_{mnf} = \left( {\rho \beta } \right)_{f} \left( {\left( {1 - \Lambda_{1} - \Lambda_{2} - \Lambda_{3} } \right) + \Lambda_{1} \left( {\frac{{\rho_{pS1} \beta_{pS1} }}{{\rho_{f} \beta_{f} }}} \right) + \Lambda_{2} \left( {\frac{{\rho_{pS2} \beta_{pS2} }}{{\rho_{f} \beta_{f} }}} \right) + \Lambda_{3} \left( {\frac{{\rho_{pS3} \beta_{pS3} }}{{\rho_{f} \beta_{f} }}} \right)} \right)$$

$$\frac{{k_{mnf} }}{{k_{f} }} = \frac{{\Lambda_{1} k_{1} + \Lambda_{2} k_{2} + \Lambda_{3} k_{3} + 2(\Lambda_{1} + \Lambda_{2} + \Lambda_{3} )k_{f} + 2(\Lambda_{1} + \Lambda_{2} + \Lambda_{3} )(\Lambda_{1} k_{1} + \Lambda_{2} k_{2} + \Lambda_{3} k_{3} ) - 2(\Lambda_{1} + \Lambda_{2} + \Lambda_{3} )^{2} k_{f} }}{{\Lambda_{1} k_{1} + \Lambda_{2} k_{2} + \Lambda_{3} k_{3} + 2(\Lambda_{1} + \Lambda_{2} + \Lambda_{3} )k_{f} - (\Lambda_{1} + \Lambda_{2} + \Lambda_{3} )(\Lambda_{1} k_{1} + \Lambda_{2} k_{2} + \Lambda_{3} k_{3} ) + (\Lambda_{1} + \Lambda_{2} + \Lambda_{3} )^{2} k_{f} }}$$

In the above expressions when $\Lambda_{3} = 0$, the properties reduce to HNFs and in the absence of $\Lambda_{3} \,\,{\text{and}}\,\,\Lambda_{2}$, the properties reduce to requisite posited NFs.

### Gradients

The important engineering factors such as $Cf_{x}$, and $Nu_{x}$ are described as follows:

13 $$Cf_{x} = \frac{{2\tau_{w} }}{{\rho_{f} U^{2} }},$$

and

14 $$Nu_{x} = \frac{{lq_{w} }}{{k_{f} \left( {T_{w} - T_{\infty } } \right)}}.$$

The expression for $Sh_{x}$ (Sherwood number) is given as:

15 $$Sh_{x} = \frac{{lq_{m} }}{{D_{B} \left( {C_{w} - C_{\infty } } \right)}}.$$

In the above equations, $\tau_{w}$, $q_{w}$ and $q_{m}$ are expressed as,

16 $$\tau_{w} = \left( {\mu_{mnf} \frac{\partial u}{{\partial y}}} \right)_{y = 0} ,\quad q_{w} = - \left( {\frac{{16\sigma^{*} T_{\infty }^{3} }}{{3k^{*} }} + k_{mnf} } \right)\left( {\frac{\partial T}{{\partial y}}} \right)_{y = 0} ,\quad q_{m} = - D_{B} \left( {\frac{\partial C}{{\partial y}}} \right)_{y = 0} .$$

By using (15) in (13–14), we get

17 $$Cf_{x} \sqrt {\text{Re}} = \frac{{f^{\prime \prime } \left( 0 \right)}}{{A_{1} }},$$

18 $$\frac{{Nu_{x} }}{{\sqrt {\text{Re}} }} = - \left( {\frac{4}{3}Rd + \frac{{k_{mnf} }}{{k_{f} }}} \right)\theta ^{\prime}\left( 0 \right),$$

19 $$\frac{{Sh_{x} }}{{\sqrt {\text{Re}} }} = - \chi^{\prime } (0),$$

where ${\text{Re}} = \frac{{lU_{*} e^{\frac{x}{l}} }}{{2\nu_{f} }}$ is called the local Reynolds number.

## Methodology

The employing computational approaches, where the simplified governing Eqs. (8–10) and BCs are addressed using the RKF-45^55^ approach and shooting procedures^56^. We turn the revised equations into a first-order system via introducing new variables. In order to transform the system of equations into first order, we will select, $f = r_{1}$, $f^{\prime } = r_{2}$, $f^{\prime \prime } = r_{3}$, $\theta = r_{4}$, $\theta^{\prime } = r_{5}$, $\chi = r_{6}$ and $\chi^{\prime } = r_{7}$. Hence, the equations become as:

20 $$r_{3}^{\prime } = - \left( {A_{1} A_{2} \left( {r_{1} r_{3} - 2\left( {r_{2} } \right)^{2} } \right) - K_{1} r_{2} \pm \lambda A_{1} A_{4} r_{4} } \right),$$

21 $$r_{5}^{\prime } = {{ - A_{3} \Pr \left( {r_{5} r_{1} - r_{2} r_{4} } \right)} \mathord{\left/ {\vphantom {{ - A_{3} \Pr \left( {r_{5} r_{1} - r_{2} r_{4} } \right)} {\left( {\frac{{k_{{mnf}} }}{{k_{f} }} + \frac{4}{3}Rd} \right)}}} \right. \kern-\nulldelimiterspace} {\left( {\frac{{k_{{mnf}} }}{{k_{f} }} + \frac{4}{3}Rd} \right)}}$$

22 $$r_{7}^{\prime } = - Sc[r_{1} r_{7} - Rr\,r_{6} ],$$

and the BCs are

23 $$\begin{gathered} r_{1} \left( 0 \right) = 0,r_{2} \left( 0 \right) = 1, \hfill \\ r_{3} \left( 0 \right) = a_{1} ,r_{4} \left( 0 \right) = 1, \hfill \\ r_{5} \left( 0 \right) = a_{2} ,r_{6} \left( 0 \right) = 1,r_{7} \left( 0 \right) = a_{3} . \hfill \\ \end{gathered}$$

To solve the IVP defined by Eqs. (20)–(22) and BCs in (23), a quantitative approach called the RKF-45 order method is applied. The shooting procedure is utilized with carefully selected error tolerance of $1/10^{6}$ and step size value of $h_{1} = 1/100$ correspondingly, to ensure that BCs at infinity are satisfied. For obtaining mathematical estimates, the built-in MATLAB program called bvp4c solver is implemented. It involves substituting parameters $K_{1} = 0.1,Rd = 1,Sc = 0.8,Rr = 0.1$ and $\lambda = \pm 0.3$, considering properties mentioned in Table 2. Graphical representation of the results generated for each constraint by varying each parameter while keeping the remaining values constant. In order to validate the numerical code for $- \theta ^{\prime}\left( 0 \right)$, a comparison is made between the findings of present study and formerly published research. The findings, as presented in Table 3, demonstrate a satisfactory level of agreement between the outcomes of the two datasets.

**Table 2:** The NPs and $H_{2} O$ thermophysical properties taken from the works of^54^.

Material	$\rho$ $\left( {{\text{kg}}/{\text{m}}^{3} } \right)$	$\beta \times 10^{ - 5}$ $\left( {{\text{K}}^{ - 1} } \right)$	$k$ $\left( {{\text{kg}}\;{\text{ms}}^{ - 3} K^{ - 1} } \right)$	$C_{p}$ $\left( {Jkg^{ - 1} K^{ - 1} } \right)$
Aluminium oxide $\left( {Al_{2} O_{3} } \right)$	3970	0.85	40	765
Silver $\left( {Ag} \right)$	6500	1.89	18	540
Titanium Oxide $\left( {TiO_{2} } \right)$	4250	0.9	8.9538	686.2
Water $\left( {H_{2} O} \right)$	997.1	21	0.613	4179

**Table 3:** Validation is performed by comparing the results with $- \theta ^{\prime}\left( 0 \right)$ values for a selection of the limiting cases.

$\Pr$	Bidin and Nazar^57^	Aziz^58^	Magyari and Keller^59^	Ishak^60^	Present study
1	0.9547	0.954785	0.954782	0.9548	0.954955
2	1.4714	–	–	1.4715	1.4714207
3	1.8691	1.869074	1.869075	1.8691	1.8690440
5	–	2.500132	2.500135	2.5001	2.5001089
10	–	3.660372	3.660379	3.6604	3.6603543

The algorithm of the RKF -45 is given below (see^55^):

$${\text{RK 4}}^{{{\text{th}}}} {\text{order}}:j_{i + 1} = + \frac{25}{{216}}m_{1} + \frac{2197}{{4104}}m_{4} + \frac{1408}{{2565}}m_{3} - \frac{1}{5}m_{5} + j_{i} .$$

$${\text{RK 5}}^{{{\text{th}}}} {\text{order}}:k_{i + 1} = \frac{2}{55}m_{6} - \frac{9}{50}m_{5} + \frac{16}{{135}}m_{1} + \frac{28561}{{56430}}m_{4} + \frac{6656}{{12825}}m_{3} + j_{i} .$$

The current methodology can be implemented by the following six steps:

$$h_{1} f\left( {n_{i} ,j_{i} } \right) = m_{1} ,$$

$$h_{1} f\left( {n_{i} + \frac{1}{4}h_{1} ,j_{i} + \frac{1}{4}m_{1} } \right) = m_{2} ,$$

$$h_{1} f\left( {n_{i} + \frac{3}{8}h_{1} ,j_{i} + \frac{3}{32}m_{1} + \frac{9}{32}m_{2} } \right) = m_{3} ,$$

$$h_{1} f\left( {n_{i} + \frac{12}{{13}}h_{1} ,j_{i} + \frac{1932}{{2147}}m_{1} - \frac{7200}{{2147}}m_{2} + \frac{7296}{{2147}}m_{3} } \right) = m_{4} ,$$

$$h_{1} f\left( {n_{i} + h_{1} ,j_{i} + \frac{439}{{216}}m_{1} - 8m_{2} + \frac{3680}{{513}}m_{3} - \frac{845}{{4104}}m_{4} } \right) = m_{5} ,$$

$$h_{1} f\left( {n_{i} + \frac{1}{2}h_{1} ,j_{i} + 2m_{2} - \frac{8}{27}m_{1} - \frac{11}{{40}}m_{5} - \frac{3544}{{2565}}m_{3} - \frac{1859}{{4104}}m_{4} } \right) = m_{6} .$$

## Results and discussion

The flow of THNF across an ESS in the presence of a permeable medium is examined in this work. The modelling takes into account the impact of chemical reactions and renewable (solar) radiation. The dimensionless equations are formed from a collection of suitable similarity variables to simplify the consider task. Several critical elements are uncovered using this technique, and their impact on the profiles is visually depicted and briefly described.

**Figure 2:**
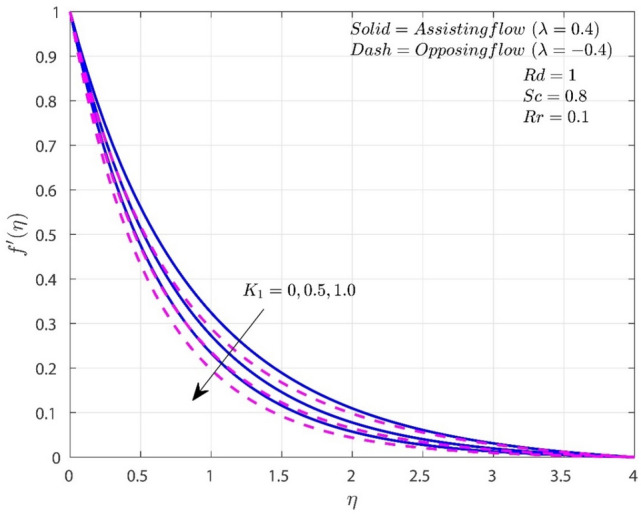
Variation of $K_{1}$ on $f^{\prime}$.

**Figure 3:**
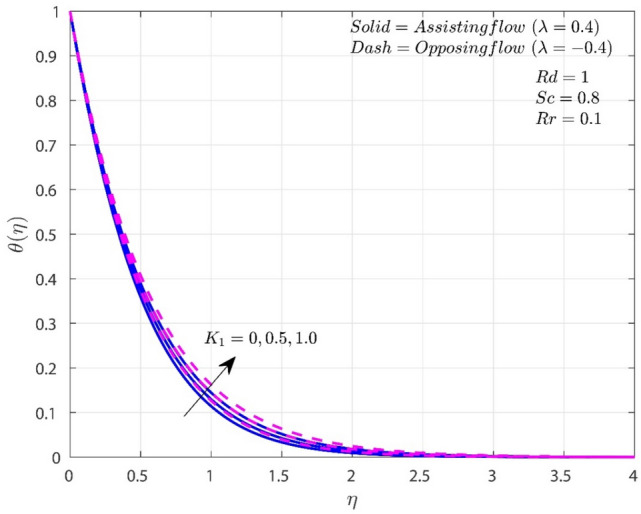
Variation of $K_{1}$ on $\theta$.

**Figure 4:**
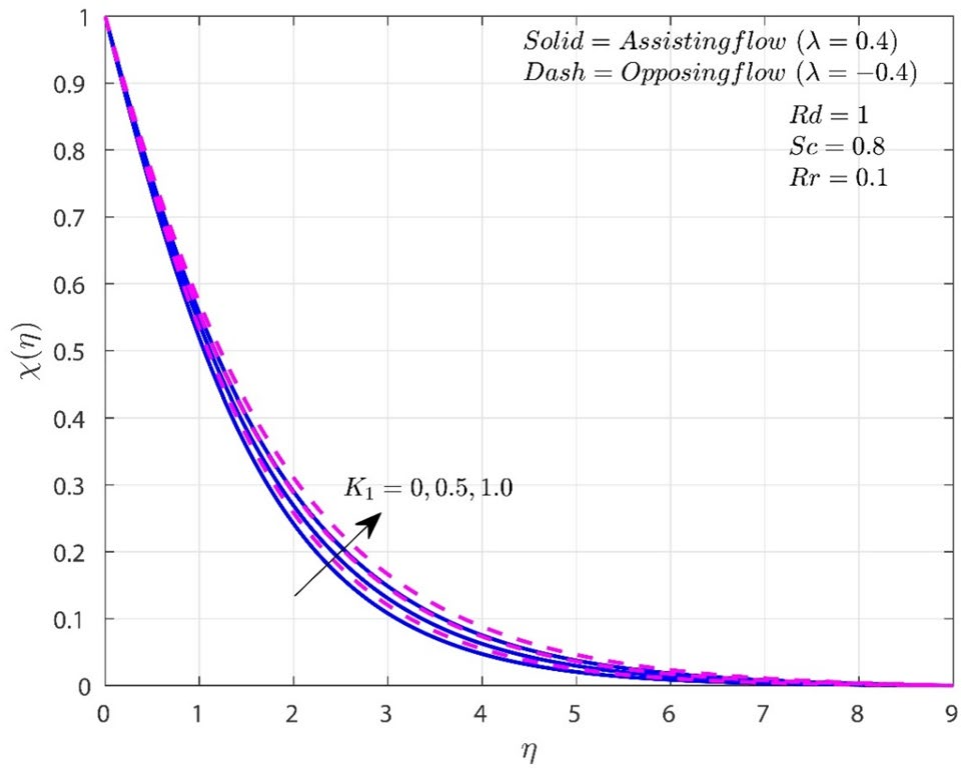
Variation of $K_{1}$ on $\chi$.

The variation of $K_{1}$ on $f^{\prime}$, $\theta$, and $\chi$ in both A-F and O-F situations are shown in Figs. 2, 3, 4. In both flow circumstances, the velocity $f^{\prime}$ falls as $K_{1}$ rises (see Fig. 2). This is happening due to the reason that the greater frictional force caused by higher porosity values, the system is more resistive and fluid flow is constrained. Potential drop in the fluid velocity is observed, and this results in higher resistance. Additionally, when $K_{1}$ rises, the fall in $f^{\prime}\left( \eta \right)$ for O-F is more noticeable than the case of A-F. In both situations, higher porosity values also lead to higher $\theta$ profiles. Temperature is improved by increasing $K_{1}$ because it thickens the thermal boundary layer (TBL). This finding is consistent with the reasonable assumption that higher $K_{1}$ improves heat retention and transmission within the liquid. In addition, when $K_{1}$ is present, heat transmission in circumstances with O-F is greater than in situations with A-F. Rise in the values of $K_{1}$ will improves the concentration for both A-F and O-F cases as shown in Fig. 4. Improvement in $K_{1}$ will improves the frictional force which results improvement in the concentration boundary layer (CBL). Consequently, the level of concentration shows significant improvement for the O-F case than the A-F case.

**Figure 5:**
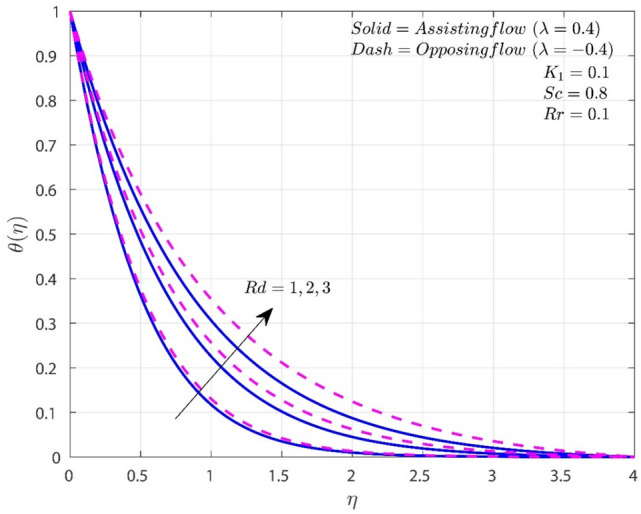
Variation of $Rd$ on $\theta$.

Figure 5 reveals the variation of $Rd$ on thermal profile. An increase in $Rd$ will enhances thermal distribution for both A-F and O-F cases. $Rd$ has an inverse relationship with the mean absorption coefficient, which drops as the value of $Rd$ increase. The buoyancy force is reduced, as a result, of conductive heat transmission, which proves to be more efficient compared to radiative heat transfer. In fact, a higher thermal dispersion is achieved with increased heat transfer to operating fluids through a higher $Rd$ value. Furthermore, gradual improvement in temperature of fluid is observed as the $Rd$ values are set to be high, and the fluid becomes more heated. Following that, heat transmission significantly increases. In the presence of $Rd$, the O-F shows high thermal distribution than A-F case.

**Figure 6:**
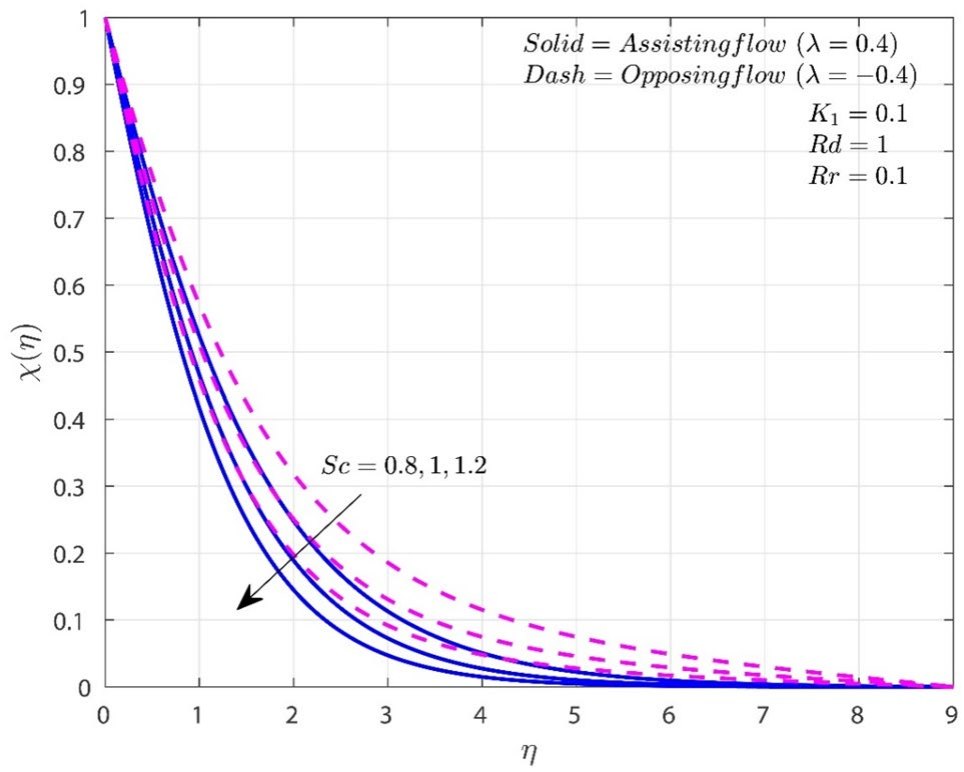
Variation of $Sc$ on $\chi$.

The impact of $Sc$ on the $\chi$ profile is observed in Fig. 6. In both scenarios, increasing $Sc$ lowers the concentration. Increased values of $Sc$ improves the mass diffusion coefficient, which lead to a reduction in concentration. This implies that the fluid's capacity to convey velocity is comparatively more effective than its capacity to convey mass or concentration. Consequently, the predominance of fluid motion supersedes mass transfer, leading to less efficient dispersion of concentration gradients arising from the origin. In comparison to the O-F situation, concentration is reduced in the A-F case.

**Figure 7:**
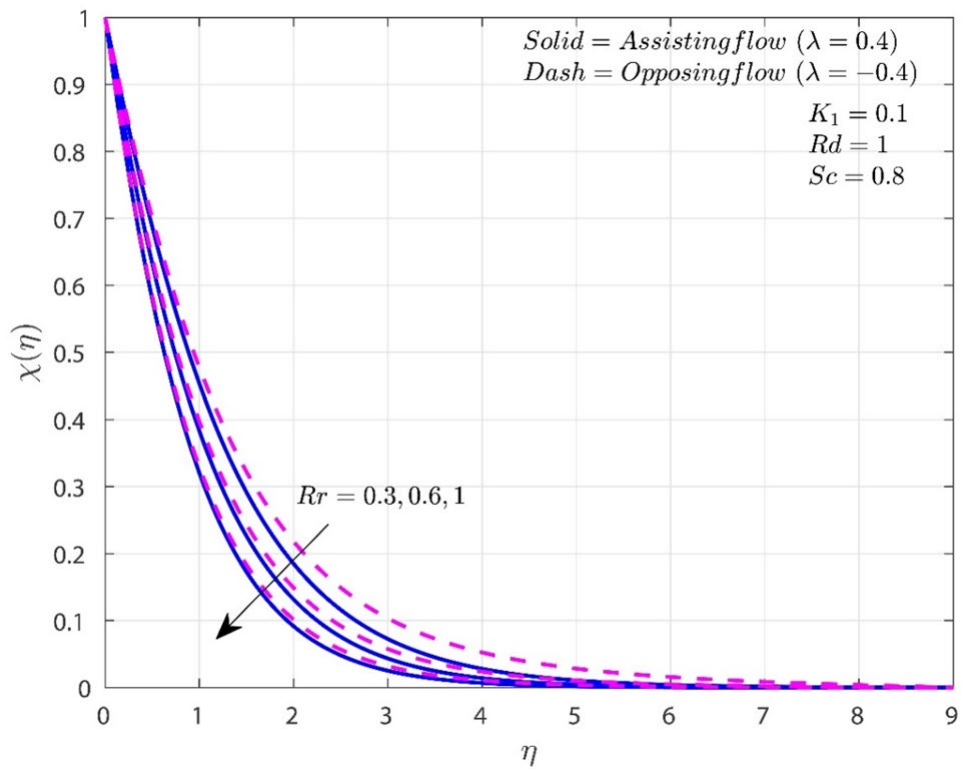
Variation of $Rr$ on $\chi$.

Figure 7 shows how the variance of $\chi$ changes with various $Rr$ values. In both situations, increasing values cause the concentration to drop. Higher $Rr$ indicates that the transformation of reactants into products is taking place at an accelerated rate causes lower CBL. When $Rr$ is present, the O-F case concentrates less than the A-F case.

**Figure 8:**
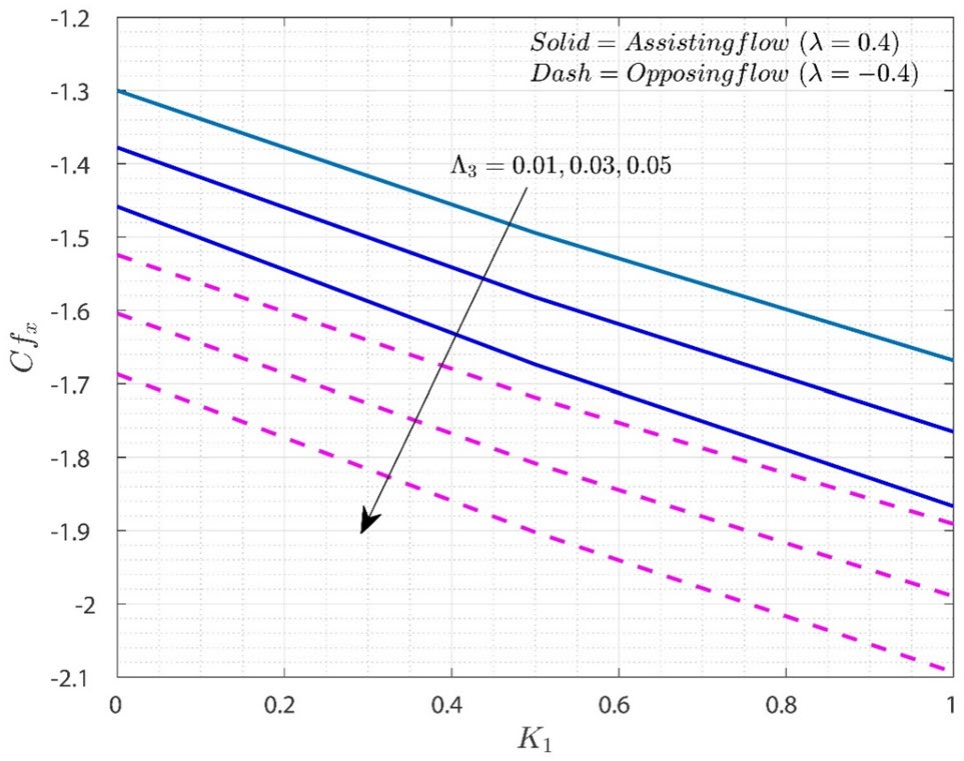
Variation of $Cf_{x}$ on $K_{1}$ for numerous values of $\Lambda_{3}$.

**Figure 9:**
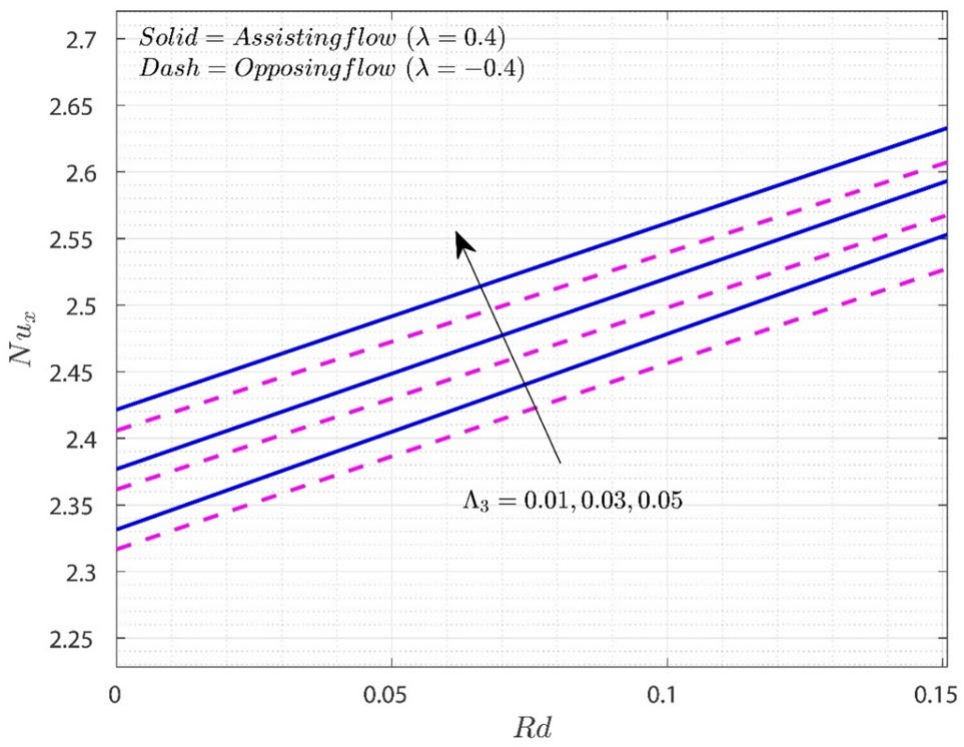
Variation of $Nu_{x}$ on $Rd$ for numerous values of $\Lambda_{3}$.

Figures 8 and 9 indicate how the key engineering interests of $Cf_{x}$ and $Nu_{x}$ vary with different dimensionless restrictions. Figure 8 depicts the fluctuation of $Cf_{x}$ on $K_{1}$ for various values of $\Lambda_{3}$ for both flow situations. The graph shows that increasing the value of $\Lambda_{3}$ decreases the friction drag force in both flows. The increase in $K_{1}$ values will resist the flow of the liquid. The addition of $K_{1}$ to the scale of $\Lambda_{3}$ enhances the MBL thickness. This results in a reduction in surface drag force. In the O-F situation, $Cf_{x}$ is much lower than in the A-F example. Figure 9 depicts the fluctuation of $Nu_{x}$ on Rd as $\Lambda_{3}$ values vary. The rise in the value of $\Lambda_{3}$ and $Rd$ will increase the thermal transfer rate.

The elevation in the values of $Rd$ improves the rate of heat transfer due to presence of $k^{*}$. Increment in $\Lambda_{3}$ also improves the TBL which results in improvement of $Nu_{x}$. $Nu_{x}$ is more in A-F case than O-F case.

**Figure 10:**
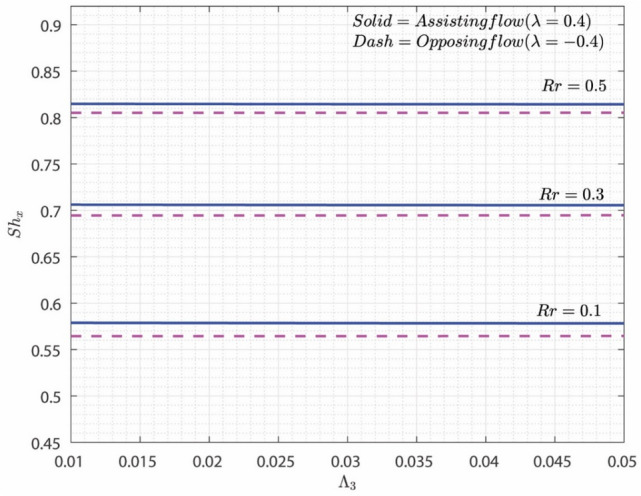
Variation of $Sh_{x}$ on $\Lambda_{3}$ for numerous values of $Rr$.

Figure 10 displays the variation of $Sh_{x}$ with change in the values of $\Lambda_{3}$ and $Rr$ for both A-F and O-F scenarios. As the values of $Rr$ escalates, the rate of transformation of reacting substances into outputs is accelerated further. On the other hand, $\Lambda_{3}$ will also improves the thickness of the CBL due to improvement in the surface area. A-F case shows greater rate of mass distribution than O-F.

Further, Table 4 shows the computational values of $Cf_{x}$,$Nu_{x}$ and $Sh_{x}$ for both A-F and O-F cases in the presence of various dimensionless constraints. Table 5 displays the change in the percentages of $Cf_{x}$,$Nu_{x}$ and $Sh_{x}$ for both A-F and O-F cases in comparison with TNFs and HNFs. The tabulated outcome shows that TNFs shows gradual improved performance in all the constraints in comparison with HNFs.

**Table 4:** Computed numerical results of $Cf_{x}$, $Nu_{x}$ and $Sh_{x}$ for several constraints which are dimensionless with $\Lambda_{1} = \Lambda_{2} = 0.01$.

$\Lambda_{3}$	$K_{1}$	$Rd$	$Rr$	$- Cf_{x}$		$Nu_{x}$		$Sh_{x}$	
0.01	0.1	1	0.1	$\lambda = 0.4$	$\lambda = - 0.4$	$\lambda = 0.4$	$\lambda = - 0.4$	$\lambda = 0.4$	$\lambda = - 0.4$
0.01				1.34078	1.56529	3.79998	3.71445	0.57882	0.56448
0.03				1.42065	1.64722	3.81106	3.72716	0.57837	0.56445
0.05				1.50363	1.73236	3.82315	3.74099	0.57815	0.56441
	0.1			1.34078	1.56529	3.79998	3.71445	0.57882	0.56448
	0.5			1.49431	1.71858	3.72790	3.63569	0.56452	0.54976
	1.0			1.66825	1.89092	3.64578	3.54638	0.54996	0.53503
		1		1.34078	1.56529	3.79998	3.71445	0.57882	0.56448
		2		1.31687	1.59441	4.71124	4.50619	0.58289	0.55889
		3		1.30019	1.61737	5.41062	5.04916	0.58625	0.55341
			0.1	1.33903	1.59390	3.80193	3.67524	0.57882	0.56448
			0.3	1.33903	1.59390	3.80193	3.67524	0.70613	0.69459
			0.5	1.33903	1.59390	3.80193	3.67524	0.81475	0.80504

**Table 5:** Comparative analysis of change in $Cf_{x} \%$,$Nu_{x} \%$ and $Sh_{x} \%$ for dimensionless constraints in the case of A-F and O-F.

$K_{1}$	$Rd$	$Rr$	$Cf_{x}$		$Nu_{x}$			$Sh_{x}$	
0.1	1	0.1	$\lambda = 0.4$	$\lambda = - 0.4$	$\lambda = 0.4$		$\lambda = - 0.4$	$\lambda = 0.4$	$\lambda = - 0.4$
0.5			2.92%	2.59%	0.12%		0.15%	0.04%	0.01%
1.0			2.87%	2.58%	0.11%		0.13%	0.04%	0.005%
1.5			2.84%	2.57%	0.09%		0.12%	0.04%	0.001%
	1.0		2.98%	2.61%	0.13%		0.16%	0.05%	0.01%
	3.0		1.74%	2.50%	14.7%		8.08%	0.50%	0.04%
	5.0		1.37%	2.41%	20.4%		15.06%	0.73%	0.11%
		0.2	2.98%	2.61%	0.13%		0.16%	0.04%	0.01%
		0.4	2.98%	2.61%	0.13%		0.16%	0.03%	0.01%
		0.6	2.98%	2.61%	0.13%		0.16%	0.02%	0.007%

The rate of change in the percentage of skin friction, Nusselt number and Sherwood number in the presence of TNF and HNF are presented in Table 5. To obtain $Cf_{x} \%$,$Nu_{x} \%$ and $Sh_{x} \%$ the formulas are given below:

24 $$Cf_{x} = \left| {\frac{{\left. {Cf_{x} } \right|_{{\Lambda_{1} = \Lambda_{2} = \Lambda_{3} = 0.01}} - \left. {Cf_{x} } \right|_{{\Lambda_{1} = \Lambda_{2} = 0.01}} }}{{\left. {Cf_{x} } \right|_{{\Lambda_{1} = \Lambda_{2} = 0.01}} }}} \right| \times 100,$$

25 $$Nu_{x} = \left| {\frac{{\left. {Nu_{x} } \right|_{{\Lambda_{1} = \Lambda_{2} = \Lambda_{3} = 0.01}} - \left. {Nu_{x} } \right|_{{\Lambda_{1} = \Lambda_{2} = 0.01}} }}{{\left. {Nu_{x} } \right|_{{\Lambda_{1} = \Lambda_{2} = 0.01}} }}} \right| \times 100,$$

26 $$Sh_{x} = \left| {\frac{{\left. {Sh_{x} } \right|_{{\Lambda_{1} = \Lambda_{2} = \Lambda_{3} = 0.01}} - \left. {Sh_{x} } \right|_{{\Lambda_{1} = \Lambda_{2} = 0.01}} }}{{\left. {Sh_{x} } \right|_{{\Lambda_{1} = \Lambda_{2} = 0.01}} }}} \right| \times 100.$$

## Final remarks

The idea of this study is to investigates the transfer of heat and mass on ESS by considering the influence of A-F and O-F with ternary hybrid nanofluid. It also incorporates T-R, chemical reaction, and porous medium. Utilizing RKF-45 the numerical solutions are found for reduced ODEs. Graphs are utilized to illustrate the important dimensionless limitations. The key findings of this work are as follows:

- The velocity profile declines with higher impacts of the porosity parameter while the temperature and concentration profiles boosted up.
- The augmentation of the solar radiation parameter decelerates the velocity, thermal distribution, and temperature gradients. This indicates that reducing thermal radiation flux decelerates the thickness of TBL while increasing the thickness of the MBL.
- For increasing thermal radiation and $\Lambda_{3}$, the heat transfer rate augmented. In general, the radiation parameter enriches the thermal conductivity, as a consequence, the heat transfer improves.
- The shear stress and mass transfer rate decelerate with superior influences of $\Lambda_{3}$ but the heat transfer rate remarkably uplifts.
- When compared to HNFs, the rate of heat distribution in THNFs goes from 0.13% to 20.4% and from 0.16% to 15.06%, respectively, for the case of A-F and as well as for the case of O-F.

The current research is relevant in several applications like capacitors, biofuel, batteries, nanomaterials, and power storage etc. By captivating the following distinct impact such as the ternary nanofluid flow over an inclined stretching/shrinking surface, mass suction/injection, convective boundary conditions, Two-phase model and Newtonian heating is also possible to consider in the extended work of this problem.

## Data Availability

The datasets used and/or analysed during the current study available from the corresponding author on reasonable request.

## List of symbols

$$T$$: Temperature (K)
$$T_{w}$$: Wall temperature (K)
$T_{\infty }$: Far-field temperature (K)
$$T_{0}$$: Reference temperature (K)
$q_{r}$: Thermal radiation heat flux
$k^{*}$: Mean absorption coefficient $\left( {{\text{m}}^{ - 1} } \right)$
$K^{*}$: Permeability of porous medium $\left( {{\text{m}}^{2} } \right)$
$K_{1}$: Porous permeability parameter
$$U,U_{*}$$: Velocity $$\left( {{\text{ms}}^{ - 1} } \right)$$
$$Nu_{x}$$: Nusselt number
$\chi$: Dimensionless concentration profile
$Rr$: Reaction rate parameter
$$D_{B}$$: Diffusion $$\left( {{\text{ms}}^{ - 2} } \right)$$
$$C$$: Concentration
$$C_{w}$$: Wall concentration
$f$: Dimensionless velocity profile
$$l$$: Length $$\left( {\text{m}} \right)$$
$$k$$: Thermal conductivity $$\left( {{\text{kg}}\;{\text{ms}}^{ - 3} {\text{K}}^{ - 1} } \right)$$
$$C_{p}$$: Specific heat $$\left( {{\text{J}}\;{\text{kg}}^{ - 1} {\text{K}}^{ - 1} } \right)$$
$$\Pr$$: Prandtl number
$$Rd$$: Radiation parameter
$u$ and $v$: Velocity components $\left( {{\text{ms}}^{ - 1} } \right)$
$$x$$ and $$y$$: Coordinates $$\left( {\text{m}} \right)$$
$$Cf_{x}$$: Skin friction
${\text{Re}}$: Local Reynolds number
$$Sc$$: Schmidt number
$g$: Acceleration due to gravity $\left( {{\text{ms}}^{ - 2} } \right)$
$$Gr$$: Grashof number
$C_{\infty }$: Far-field concentration
$$C_{0}$$: Reference concentration

## Greek symbols

$$\Psi$$: Stream function
$$\beta$$: Thermal expansion $$\left( {{\text{K}}^{ - 1} } \right)$$
$$\nu$$: Kinematic viscosity $$\left( {{\text{m}}^{2} {\text{s}}^{ - 1} } \right)$$
$\sigma^{*}$: Stefan–Boltzmann constant $\left( {{\text{kg}}\;{\text{s}}^{ - 3} {\text{K}}^{ - 4} } \right)$
$$\mu$$: Dynamic viscosity $$\left( {{\text{kg}}\,{\text{m}}^{ - 1} {\text{s}}^{ - 1} } \right)$$
$\delta_{r} ,\delta_{0}$: Variable chemical rate (${\text{s}}^{ - 1}$)
$\lambda$: Mixed convection parameter
$\theta$: Dimensionless temperature profile
$$\eta$$: Similarity variable
$\Lambda$: Solid volume fraction
$$\rho$$: Density $$\left( {{\text{kg}}\,{\text{m}}^{ - 3} } \right)$$

## Subscripts

$$mnf$$: Modified nanofluid
$$nf$$: Nanofluid
$$S1,S2,S3$$: Solid particles
$$hnf$$: Hybrid nanofluid
$$f$$: Fluid
